# Conspecific Scaffold‐Level Genome Assembly Outperforms Heterospecific Chromosome‐Level Assemblies for Assessing Genetic Indicators in a Threatened Marine Fish

**DOI:** 10.1111/eva.70247

**Published:** 2026-05-05

**Authors:** Mannda Ndou, Warren Mason Potts, Tuan Anh Duong, Peter Rodja Teske, Amber‐Robyn Childs, Romina Henriques

**Affiliations:** ^1^ Department of Biochemistry, Genetics and Microbiology University of Pretoria Pretoria South Africa; ^2^ Department of Ichthyology and Fisheries Sciences Rhodes University Makhanda South Africa; ^3^ Forestry and Agricultural Biotechnology Institute University of Pretoria Pretoria South Africa; ^4^ Centre for Ecological Genomics and Wildlife Conservation, Department of Zoology University of Johannesburg Auckland Park South Africa

**Keywords:** genetic indicator metrics, non‐model species, single nucleotide polymorphism (SNPs), whole genome sequencing

## Abstract

Understanding the genomic architecture of species of conservation concern is essential for fostering effective conservation initiatives. Current biodiversity assessment approaches increasingly incorporate genetic metrics to evaluate the status of species and populations of conservation interest. However, due to the limited availability of conspecific genomes for most non‐model species, previous studies have often depended on heterospecific genomes. This approach has been shown to significantly impact the precision of genetic metrics, resulting in inaccurate measurements and insights. However, it is currently unknown what the impact of using non‐species‐specific genomes is for the determination of genetic indicators in vulnerable marine fishes, such as red roman ( *Chrysoblephus laticeps* ). Red roman is a southern African endemic species, which, due to overexploitation, is currently considered Near Threatened on the International Union for Conservation of Nature (IUCN) Red List. Recent studies have shown significant life history and physiological differences between exploited and protected populations. Here, we present the first high‐quality scaffold‐level genome assembly and annotations for the red roman, using a combination of Oxford Nanopore and Illumina sequencing, and compared key genetic indicators (diversity, population structure and effective population size) obtained from reads mapped to the genomes of other Sparidae species, with well‐established genomic resources. The final assembly had 1263 scaffolds, a total length of 758 Mb, with a scaffold N50 of 5.89 Mb and Benchmarking Universal Single‐Copy Orthologs (BUSCO) completeness of 99.30%. As expected, comparative analyses with genomes of different sparid species revealed enhanced read alignment, genotyping accuracy and single nucleotide polymorphism (SNP) retention after filtering. Furthermore, there were significant overall differences across the genomes for the measures of observed heterozygosity (*H*_O_), nucleotide diversity (*π*), Tajima's *D*, *F*_ST_ and estimates of effective population size (*N*_e_), with different genomes presenting different (and sometimes contrasting) genetic indicators metrics and, consequently, demographic histories for red roman. Our study not only significantly improved genomic resources for genomic conservation analyses in *C. laticeps* , but most importantly highlighted the importance of species‐specific reference genomes for accurate evolutionary and conservation inference in highly variable marine fishes.

## 1 Introduction

Genetics and genomic datasets are pivotal for understanding species‐environment interactions, population history, genetic diversity, the basis of adaptation and predicting species' resilience to changing environmental conditions (Bay et al. 2017; Kardos et al. 2021). Although it has been argued that genome‐wide genetic variation is of limited importance for conservation actions due to its weak correlation with conservation status (Teixeira and Huber 2021), the broad literature strongly supports the importance of including genome‐wide genetic diversity metrics for the effective conservation of threatened species (DeWoody et al. 2021; Kardos et al. 2021; Kumar 2023; Nielsen et al. 2023; Ramanatha Rao and Hodgkin 2002; Salgotra and Chauhan 2023). Despite the concept that genomic data should be integrated into conservation planning is not necessarily new (Luikart et al. 2003), its full uptake has yet to be achieved (Nielsen et al. 2023), and a ‘conservation genomics gap’ remains, with the importance of genomic data recognised but not yet routinely implemented in conservation actions (Hogg 2024).

As the cost of whole genome sequencing continues to decline (Worley et al. 2017), conservation genomics is increasingly adopting the use of high‐resolution genome‐wide single nucleotide polymorphisms (SNPs) over the use of traditional genetic markers such as mitochondrial DNA (mtDNA) and nuclear microsatellites, to develop conservation strategies for threatened species (Galla et al. 2018). Mitochondrial and microsatellite markers have been effectively utilised in conservation genetics, e.g., genetic connectivity of red roman populations between marine protected areas (MPAs) and exploited areas by Teske et al. (2010). However, the mitochondrial genome is haploid, uniparental (maternally inherited), and has an effective population size (*N*_e_) approximately one‐quarter of the nuclear genome (Allendorf et al. 2012; Galtier et al. 2009; Shokolenko and Alexeyev 2022). Therefore, it can bias or underestimate total genetic variation, especially if paternal lineages differ significantly. Similarly, microsatellite markers are typically small, sparse and unevenly distributed across the genome, which may limit their applicability and misrepresent true genetic variation (Jorgenson and Witte 2007; Väli et al. 2008). In contrast, genome‐wide SNPs are biparentally inherited (diploid) (Lynch and Walsh 1998) and represent the overall population diversity (Davey et al. 2011; Yirgu et al. 2023). They offer a more accurate estimation of genetic diversity (Supple and Shapiro 2018), while also possessing higher statistical power to infer signatures of selection and local adaptation, even in the face of gene flow (Fuentes‐Pardo and Ruzzante 2017).

While SNPs can be discovered and genotyped de novo using reduced representation sequencing methods such as genotyping‐by‐sequencing (GBS) (Elshire et al. 2011), double digest Restriction‐site Associated DNA Sequencing (ddRAD‐seq) (Peterson et al. 2012), specific locus amplified fragment sequencing (SLAF‐seq) (Sun et al. 2013) and RNA sequencing (Lopez‐Maestre et al. 2016), reference‐based SNP discovery offers many advantages. This encompasses detection accuracy at low sequencing depth, enhanced ability to pinpoint genomic location, improved computational efficiency, higher statistical power to identify sequence contamination and higher precision and resolution for downstream population genetics analyses (Davey et al. 2011; Ilut et al. 2014; Oyler‐McCance et al. 2016). However, a considerable number of non‐model species still lack reference genomes, and the majority of the genomes available have not been annotated and are often poor‐quality scaffold‐level assemblies, with only a few chromosome‐level assemblies (Lewin et al. 2022). In the past 25 years, there has been a large increase in the number of genome assemblies generated and published in genomic research databases, but there are still knowledge gaps biased towards both taxonomic and geographical representation (Hotaling et al. 2021). In particular, most of the published genomes of non‐model species are from East Asia, North America and Europe, where the sequencing platforms are well established (Hotaling et al. 2021). Poorly resourced geographical regions, mostly located in the Global South, are thus particularly disadvantaged in terms of genomic data production (de Jong et al. 2024), while grappling with fast biodiversity loss. In particular, despite the wide biodiversity of marine life, genomic representation of marine species is comparatively small, with only 3.5% of species, 12% of genera and 41% of marine vertebrate families sequenced (de Jong et al. 2024). Due to the paucity of published genomes for most genera that do not include any model species, researchers often have no choice but to use reference genomes from different genera (Deng et al. 2022; Galla et al. 2018).

Genetic indicators are progressively recognised as reliable and cost‐effective tools in conservation, providing insights into species' genetic diversity, connectivity patterns and overall viability (Hoban et al. 2024; McLaughlin et al. 2025). They are measurable metrics that play a crucial role in the assessment, maintenance and monitoring of genetic diversity within populations (Brown 2008; Pierson et al. 2015). Observed heterozygosity (*H*_O_) (Nei 1973), nucleotide diversity (π) (Nei 1987; Nei and Li 1979), Tajima's *D* (Tajima 1989a, 1989b), population differentiation (*F*_ST_) (Weir and Cockerham 1984; Wright 1965, 1949) and effective population size (*N*_e_) (Wright 1931) are among the most commonly used and fundamental genetic indicator metrics for conservation, not only for measuring genetic diversity and connectivity, but also for inferring evolutionary forces and demographic history within and among populations (Ellegren and Galtier 2016; Frankham 2005; Hoban et al. 2022; Meirmans and Hedrick 2011; Toro and Caballero 2005). However, the choice of the reference genome used for conservation studies can introduce bias in downstream analyses, and consequently in these estimators, which could be caused by several factors, ranging from poor mapping, inaccurate genotyping, or a large phylogenetic distance between the study species and the species whose genome is used as the reference (Bohling 2020; Thorburn et al. 2023; Valiente‐Mullor et al. 2021). Therefore, although heterospecific reference genomes (i.e., those from different species) can still be used to identify SNPs in the species of interest (Galla et al. 2018), the accuracy of the measures of the processes shaping the evolution of organisms may be limited (Akopyan et al. 2024). In particular, the use of heterospecific genomes can downward‐bias estimates of genetic diversity and *N*_e_ (Akopyan et al. 2024), two of the most important genetic indicators used in conservation biology (Fedorca et al. 2024; Hoban et al. 2023; Waples 2002). In fact, even genomes from conspecific individuals that occur in different geographical regions and that constitute distinct evolutionary lineages from the study species may adversely impact mapping and genotyping rates, as well as inferences of local adaptation, as demonstrated for the three‐spined stickleback (Thorburn et al. 2023). In this study, Thorburn et al. (2023) showed that although the distributions of the metrics of diversity (π), differentiation (*F*_ST_) and demography (Tajima's *D*) were found to be comparable between the local genome vs. the genome from another geographical location, they nonetheless revealed distinct sets of outlier genes. Similarly, Prasad et al. (2022) investigated the impact of phylogenetic distance between a reference genome and a target species on heterozygosity and demographic inference in whales and birds. Their findings indicated that reference genomes exhibiting more than 3% divergence from the target species resulted in increased discrepancies in heterozygosity estimates as the phylogenetic distance increased. Furthermore, when distant genomes are further associated with low‐quality sequencing, the precision of genetic metrics is adversely affected (Bryc et al. 2013; Nevado et al. 2014; Prasad et al. 2022). This includes a reduction in the number of SNPs identified, a less accurate inbreeding coefficient (*F*_IS_), diminished values for π and *F*_ST_ and unreliable Principal Component Analyses (PCA) and admixture analyses (Deng et al. 2022).

A growing body of knowledge recognises the need to use conspecific genomes to increase the accuracy and precision of genetic metrics. To the best of our knowledge, no previous studies have compared the impact of using non‐species‐specific genomes in estimates of diversity and population structure metrics in genetically highly diverse and endangered sparid fish species such as the southern African endemic red roman, *Chrysoblephus laticeps* (Valenciennes, 1830). Due to their life‐history features such as philopatry, late maturity and longevity, sparids are particularly vulnerable to exploitation, and population declines have been reported for many species within southern Africa (Griffiths 2000; Mann 2013). Red roman has a distribution range extending from Namibia to Port St Johns, in eastern South Africa, but its centre of abundance is limited from False Bay to the Kei River, in South Africa (Griffiths and Wilke 2002; Heemstra and Heemstra 2004). Due to fishing pressures, the species is considered Near Threatened on the International Union for Conservation of Nature (IUCN) Red List (Mann et al. 2014), and recent studies have shown significant life history (Götz et al. 2008) and physiological (Duncan et al. 2019) differences between exploited and protected populations, i.e., populations located in no‐take Marine Protected Areas (MPAs). This makes the red roman an ideal candidate to investigate the impact of using non‐species‐specific vs. species‐specific genomes in genetic indicators, important to understand the impacts of exploitation vs. non‐exploitation in genetic diversity, connectivity and *N*_*e*_ to assist with ongoing conservation efforts. Research indicates that MPAs in South Africa contain populations with higher physiological diversity of red roman individuals when compared to regions subjected to exploitation (Duncan et al. 2019). However, the implications of these MPAs on the genetic diversity of this species remain to be investigated. In this context, our present work aims to evaluate how the first annotated scaffold‐level genome assembly produced for red roman compares to published scaffold‐ and chromosome‐level genomes of phylogenetically different sparid species in terms of genotyping, SNP discovery, SNP retention after filtering and measures of genetic indicators for samples collected from an MPA and an exploited area. We hypothesise that the use of a species‐specific genome will result in improved genotyping, SNP discovery and retention after filtering and better estimation of genetic diversity and differentiation, compared to the genome assemblies of the other sparid species. Our findings support this hypothesis and reveal that the phylogenetic distance of genomes relative to the study samples can strongly impact not only the value of the genetic indicators, but also the underlying inferred demographic history of the species, emphasising the significance of generating species‐specific reference genomes for improved conservation genomic studies.

## 2 Methods

### 2.1 Genome DNA Extraction, Sequencing, Assembly and Annotation

Sampling permits were obtained from the Department of Forestry, Fisheries and the Environment, Pretoria, South Africa (RES2024/049) and the Department of Agriculture, Land Reform and Rural Development, Pretoria, South Africa (12/11/1/1 (2507SR)), while ethical clearance for the research involving animals was granted by the Faculty of Natural and Agricultural Sciences Research Ethics Committee and Faculty of Veterinary Science Animal Ethics Committee (NAS177/2022) of the University of Pretoria, Pretoria, South Africa, as well as the Animal Ethics Committee (DIFS152018 and 2023‐7368‐7776) of Rhodes University, Makhanda, South Africa. Muscle, heart, liver and brain tissues were harvested from an adult red roman caught in Goukamma MPA (Western Cape, South Africa) and humanely euthanised at the Department of Ichthyology and Fisheries Sciences (Rhodes University, Makhanda, South Africa), following their best practices. Samples were immediately frozen in liquid nitrogen and stored at −80°C until DNA and RNA extractions commenced. High molecular weight (HMW) DNA was extracted from the frozen and pulverised heart sample using the Macherey‐Nagel HMW DNA Extraction Kit (Macherey‐Nagel, Düren, Germany), following the manufacturer's protocol. DNA concentration was assessed with Qubit (Thermo Fisher Scientific, Waltham, MA, USA), and the integrity was evaluated on a 2% agarose gel stained with GelRed (Biotium, Fremont, CA, USA), as well as in a TapeStation (Agilent Technologies, Santa Clara, CA, USA). The genome was sequenced by the University of Pretoria Genomics Laboratory (UPGL) DIPLOMICS cluster, Pretoria, South Africa, using a PromethION 2 Solo device to generate Oxford Nanopore long reads. In addition, HMW DNA was also sequenced using Illumina Whole Genome Resequencing TruSeq DNA PCR‐Free library (paired‐end 151 bp) at 100× depth, on an Illumina NovaSeq 6000 to assist with draft assembly polishing. To assist with genome annotation, RNA was extracted from brain (BRNA), liver (LRNA) and muscle (MRNA) tissues using the Macherey‐Nagel NucleoSpin RNA extraction kit (Macherey‐Nagel, Düren, Germany) following the manufacturer's protocol. The RNA was sequenced at 100× depth by Transcriptome Resequencing using Illumina TrueSeq Stranded mRNA library preparation with poly‐A mRNA enrichment (paired‐end 151 bp). Both the DNA and RNA for genome polishing and annotation were sequenced at Macrogen Europe (Amsterdam, The Netherlands). All material was destroyed after sequencing, and data were transferred to the University of Pretoria.

The quality of the Oxford Nanopore long reads was evaluated from the PromethION 2 Solo sequencing final report, and the quality of the short DNA and RNA reads was evaluated using FastQC v0.12.1 (Andrews 2010). Adapter sequences from the DNA long reads were trimmed using Porechop v0.2.4 (Bonenfant et al. 2022), while adapter sequences and low‐quality bases below an average Phred quality score threshold of 28 for the DNA and RNA short reads were trimmed using Trimmomatic v0.36 (Bolger et al. 2014). The genome was assembled using the long reads to scaffold level using the Flye v2.9 genome assembly pipeline (Kolmogorov et al. 2019), with an estimated genome length of 876 Mb based on a published genome of a related species ( *Pagrus major* , NCBI Accession GCA_002897255.1; Sawayama et al. 2017). The filtered DNA short reads were then used to polish the draft assembly using Polypolish v0.6.0 (Wick and Holt 2022) and the Polca (Zimin and Salzberg 2020) function in MaSuRCA v4.1.1 (Zimin et al. 2013). This approach was based on the methodology described by Wick and Holt (2022), which indicated that optimal draft genome assembly polishing can be achieved by applying both polishers in succession. RepeatModeler v2.0.1 (Flynn et al. 2020) was used to identify repeat elements in the genome, and RepeatMasker v4.0.7 (Smit et al. 2017) was used to soft‐mask the repeat regions with a repeat library obtained from RepeatModeler. Soft‐masked assembly Benchmarking Universal Single‐Copy Orthologs (BUSCO) completeness (i.e., percentage of complete (C), fragmented (F), duplicated (D) and missing Single‐Copy Orthologs (M)), as well as statistics (i.e., total length of assembly, number of scaffolds, number of contigs, scaffold and contigs N50) were assessed using BUSCO v5.7.1 (Simão et al. 2015; Manni et al. 2021) based on the Actinopterygii_odb10 dataset. Additional assembly statistics (i.e., longest contig, N90, L50, L90 and GC content) were assessed using Quast v5.2.0 (Gurevich et al. 2013). The masked genome was structurally annotated using Braker v3.0.8 (Gabriel et al. 2024), which integrates gene models' predictions from Augustus v3.5.0 (Stanke et al. 2006) and GeneMark v4 (Besemer and Borodovsky 2005), incorporating RNA sequence data as supporting gene evidence, as referenced in Kim and Kim (2022). The protein output from Braker structural annotations was functionally annotated using eggNOG‐mapper v2 (Cantalapiedra et al. 2021), InterProScan v5.62–94.0 (Jones et al. 2014) and BLASTp v2.7.1 (Camacho et al. 2009). The assembly quality assessment and visualisation of potential contaminations were performed using BlobTools v4.4.0 (Laetsch and Blaxter 2017). The structural annotations were assessed to determine the extent of annotated organisms' gene space using BUSCO v5.7.1 based on the Actinopterygii_odb10 dataset. For the isolation of the mtDNA sequence (to be used for the mitogenome phylogenetic tree) from the nuclear DNA, the genome was blasted against the published mtDNA genome of *P. major* (NCBI accession NC_003196) using BLASTn v2.12.0 (Camacho et al. 2009), and the identified contigs were classified as mtDNA of the red roman.

To assess the relationship among red roman and the three sparid species ( *Pagrus major* (NCBI Accession GCA_002897255.1), *Sparus aurata* (NCBI Accession GCA_900880675.2) and *Acanthopagrus latus* (NCBI Accession GCF_904848185.1)) used in this study for comparative purposes, their mitogenomes (chosen due to their small sizes which make them computationally efficient) were aligned using MAFFT v7.453 (Katoh and Standley 2013), and the phylogeny and pairwise genetic distances were estimated using IQ‐TREE v2.1.2 (Minh et al. 2020). The treefile output from IQ‐TREE was used to plot and annotate a phylogenetic tree (midpoint rooted) using Figtree v1.4.4 (Rambaut 2018).

For DNA extraction of study samples, fin clips of ten unrelated red roman individuals, stored in 96% ethanol at the Department of Ichthyology and Fisheries Sciences Laboratory (Rhodes University, Makhanda, South Africa), were used. Samples were obtained from two locations in South Africa: five (TK1, TK2, TK3, TK4, TK5) from Tsitsikamma MPA, in the Western Cape, and five (NH1, NH2, NH3, NH4, NH5) from Gqeberha (exploited area), in the Eastern Cape. DNA was extracted using the EZNA DNA Extraction Kit (Omega Bio‐Tek, Norcross, GA, USA) following the manufacturer's protocol, and the quality and quantity of extracted DNA were assessed using GelRed (Biotium, Fremont, CA, USA), Nanodrop 2000 (Thermo Fisher Scientific, Wilmington, DE, USA) and Qubit (Thermo Fisher Scientific, Waltham, MA, USA). DNA samples were sent for paired‐end (150 bp) whole genome resequencing, with medium coverage (10–15×), using a DNBSeq platform at BGI (Hong Kong, China). The resulting raw reads underwent quality assessment using FastQC and were trimmed with Trimmomatic as described for DNA and RNA methodology for genome polishing and annotation.

### 2.2 Mapping and SNP Calling Comparison Among Different Types of Reference Genomes

In order to evaluate how different assembly levels of reference genomes (in terms of completeness) and their phylogenetic relationship might influence inference of genetic diversity, population differentiation and *N*_e_ in a threatened marine fish, the trimmed sequences from the 10 red roman individuals were mapped to: (i) the scaffold‐level genome of red roman generated in this study; (ii) the scaffold‐level genome of the *P. major* (NCBI Accession GCA_002897255.1; Sawayama et al. 2017); the chromosome‐level genome of the *Sparus aurata* (NCBI Accession GCA_900880675.2); and (iv) the chromosome‐level genome of *Acanthopagrus latus* (NCBI Accession GCF_904848185.1). Short read mapping was performed using BWA‐mem v0.7.17 (Li and Durbin 2010), and the resulting alignment files underwent collation, fixmating, sorting by position, duplication marking, with the addition of read groups using Samtools v1.9 (Li et al. 2009), before being used for SNPs calling. The first metric of accuracy across the four reference genomes was mapping success, interpreted as the percentages of mapped and properly paired reads for all 10 samples across the four reference genomes using Samtools flagstats, with statistical significance across the genomes measured using a Kruskal‐Wallis test (Kruskal and Wallis 1952), followed by Dunn's test (Dunn 1964) between pairs of genomes, with *p*‐values adjusted with the Benjamini‐Hochberg correction method (Benjamini and Hochberg 1995) in R v4.4.3 (R Core Team 2024).

SNP calling was conducted using BCFtools v1.20 (Li 2011), employing an mpileup approach. Two VCF files were generated per genome, one containing variable sites only and the other containing both variable and invariable sites. In particular, the VCF files with variable and invariable sites were generated solely for Tajima's *D* and π estimation as suggested by Korunes and Samuk (2021), who showed that an accurate and unbiased estimation of π can only be made from a file with both variable and invariable sites. The VCF files with only variable sites were filtered using BCFtools for biallelic SNPs, no missing data, a Minor Allele Frequency (MAF) no less than 0.2, a minimum coverage depth of 3 reads per genotype position, Linkage Disequilibrium (LD) of *r* ^2^ > 0.8, and no insertion and deletion mutations (INDELS), while the VCF file with both variable and invariable sites was similarly filtered, except for retaining biallelic genotypes (instead of SNPs). Both the VCF files per genome were finally filtered for reference‐specific SNPs that were fixed for the alternate allele throughout the samples and heterozygosity excess greater than 0.9 to remove heterozygous genotypes potentially caused by sequencing error. All filtering steps were conducted using BCFtools v1.20 and VCFtools v0.1.17 (Danecek et al. 2011). A second metric of accuracy across the four genomes was estimated as the number of records and SNPs recovered and retained after filtering, with differences across genomes assessed based on the percentage and fold of reduction between the before and after filtering records and SNPs. Such a descriptive trend analysis was performed in this case because the input lacked replication of values that would qualify it for a statistical significance test.

### 2.3 Measures of Genetic Diversity, Differentiation and Effective Population Size

**FIGURE 1:**
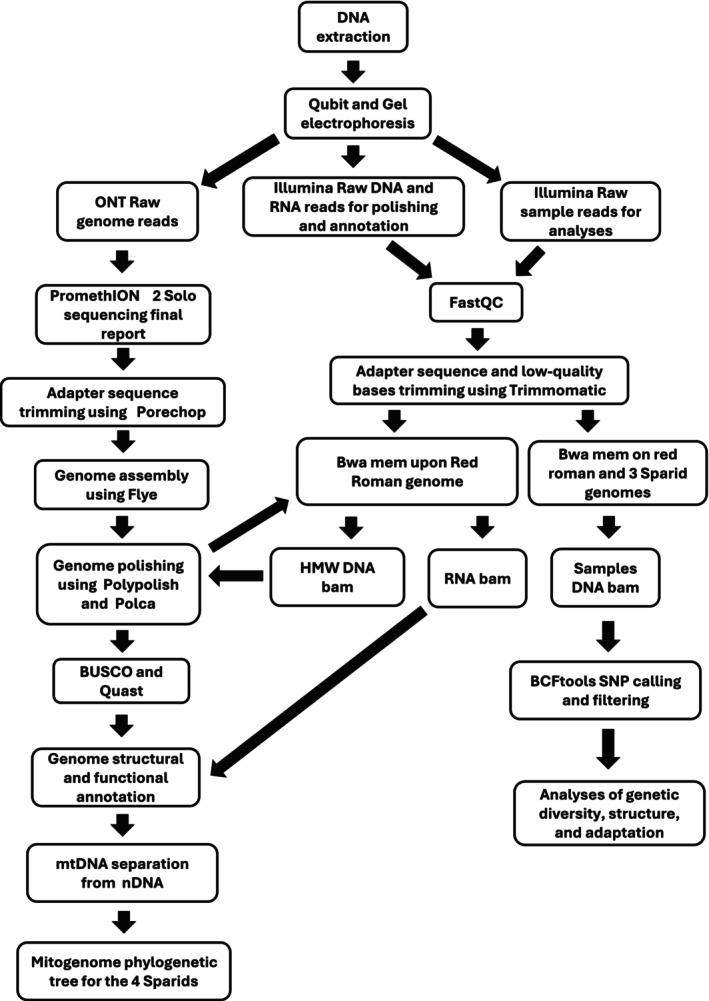
A diagram depicting the methodology pipeline used in this study, from DNA extraction to the analysis of the genetic metrics.

Genetic indicators are fundamental for monitoring vulnerability in populations, and three main metrics are used to report on the status of species: diversity, connectivity and *N*_*e*_ (Hoban et al. 2024). To test how different types of reference genomes might impact these estimators, we calculated: (i) genome‐wide genetic diversity for MPA and non‐MPA sites, and across genomes, including heterozygosity per site (for the variable sites VCF only), π and Tajima's *D* (for the variable & invariable sites VCF file only) in VCFtools; (ii) pairwise *F*_ST_ levels in VCFtools (variable sites VCF only) and (iii) genome‐based *N*_e_ (Wright 1931) through LD (Hill 1981), Heterozygosity Excess (Cornuet and Luikart 1996), and Molecular Coancestry (Nomura 2008) methods in NeEstimator v2.1 (Do et al. 2014). Except for *N*_e_, which was evaluated based on genomes rather than populations to maximise sample size and employs confidence intervals to gauge the reliability of the estimates, the statistical significance of differences observed between the two sampling sites (Wilcoxon test; Wilcoxon 1945), across genomes (Kruskal‐Wallis test), and for pairwise reference genome types (Dunn's test), was conducted in R v4.4.3. The overall methodology approach is summarised in Figure 1.

## 3 Results

### 3.1 Genome Assembly, Annotation and Mitogenome Isolation

**FIGURE 2:**
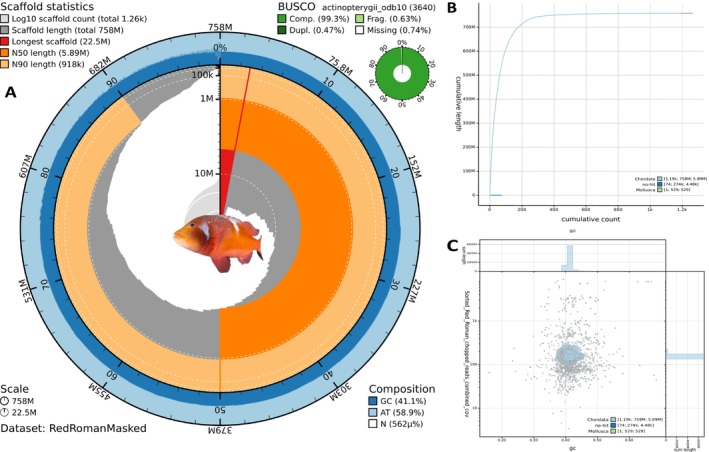
Blobtools plots summarising the *C. laticeps* genome assembly statistics, including: (A) Snail plot visualisation of the length and composition of the genome, with the BUSCO scores at the top right; (B) Cumulative graph depicting a relationship between the number of scaffolds and their length towards the genome's total size, (C) Blob plot visualises the amount of GC content and coverage depth for the assembly scaffolds.

The Oxford Nanopore sequencing generated 22.63 million reads and retrieved 124.29 Gb, with an estimated N50 of 10.43 kb. Genome assembly resulted in a total genome length of 758 Mb, with 161× coverage depth (Figure S1), 1263 scaffolds and 1306 contigs. The 100× depth HMW short‐read DNA sequencing for genome polishing generated 880 million reads and 133 Gbp, whereas the 100× depth RNA sequencing for genome annotation generated, per tissue, 60 million reads and 9Gbp for BRNA, 66 million reads and 9Gbp for LRNA and 59 million reads and 8 Gbp for MRNA. After polishing the assembly using short‐read sequences and soft‐masking its repeats, the longest scaffold was 22.5 Mb, with an N50 of 5.89 Mb, N90 of 918 KB, L50 of 36 and L90 of 153 (Figure 1A). The whole genome had an average composition of 41.1% GC. From a total of 3640 BUSCO genes searched (*n*), 99.3% were complete (C), 98.8% were complete and single‐copy (S), 0.5% were complete and duplicated (D), 0.6% were fragmented (F) and 0.1% were missing (M). The cumulative graph (Figure 2B) and blob plot (in addition to the genome coverage depth visualisation and GC content described above, Figure 2C) indicated that approximately 99.96% of the genome's total length of 758 Mb, comprising 1.19 k sequences with an N50 length of 5.89 Mb, was assigned to the phylum Chordata, while approximately 0.0007% (1 sequence (contig 78) with a cumulative length and total length of 529) was attributed to Mollusca. Notably, about 0.04% (74 sequences with a total length of 274 k and an N50 length of 4.48 k) did not have a match. While the attribution of contig 78 to Mollusca raised contamination concerns, BLASTn results from the NCBI nucleotide collection attributed the contig to sparid species, as expected, with the top match to *Pagrus pagrus* (Figure S2). Therefore, this contig was not removed from the dataset because its assignment to Mollusca might reflect the influence of multiple weaker cross‐hits to conserved loci rather than a genuine Mollusca contaminant. The 0.04% that did not have any match could imply the generation of a new sequence for the red roman that does not exist in the database.

Structural annotation using Braker3 predicted a total of 39,218 protein‐coding genes and 46,352 transcripts. Functional annotations recovered 25,547 genes (65.14%) and 32,407 transcripts (69.91%) by eggNOG‐mapper, 349,78 genes (89.19%) and 42,013 transcripts (90.64%) by InterProScan and 24,444 genes (62.33%) and 31,206 transcripts (67.32%) by BLASTp. A BUSCO analysis conducted on the structural annotation evaluated a total of 3640 BUSCO groups, showing that 97.1% were categorised as Complete, 78.7% as Single copy, 18.4% as Duplicated, 1.6% as Fragmented and 1.3% as Missing.

**FIGURE 3:**
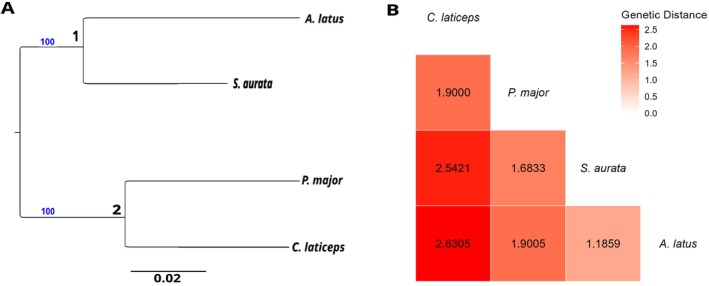
Phylogenetic relationships and genetic divergence of the four sparid species. (A) Maximum likelihood mitogenome phylogeny reconstructed using the TIM2+F+I substitution model (selected via BIC). The scale bar represents 0.02 substitutions per site. Values shown in blue on the clades represent Ultrafast Bootstrap (UFB) support percentages. (B) Heatmap showing pairwise genetic distances between the four species.

The BLASTn results of the genome against the mtDNA of *P. major* identified contig_2018 and contig_2019 as hits (Table S1), with a total length of 16,910 bases, as expected. Phylogenetic analyses based on the mitogenomes of the four species identified two clades, both with 100% bootstrap support, of which *A. latus* and *S. aurata* formed clade 1, and *P. major* and *C. laticeps* formed clade 2 (Figure 3A), as expected from the published multi‐locus phylogenetic tree of the Sparidae family (Santini et al. 2014, Figure S3). This phylogeny was further supported by estimates of pairwise genetic distances, where the relatedness of the three sparid species relative to *C. laticeps* was lower for *P. major* (in clade 1 with *C. laticeps* ), followed by *S. aurata* and *A. latus* , both in clade 2 (Figure 3B).

### 3.2 Mapping and SNP Calling

**FIGURE 4:**
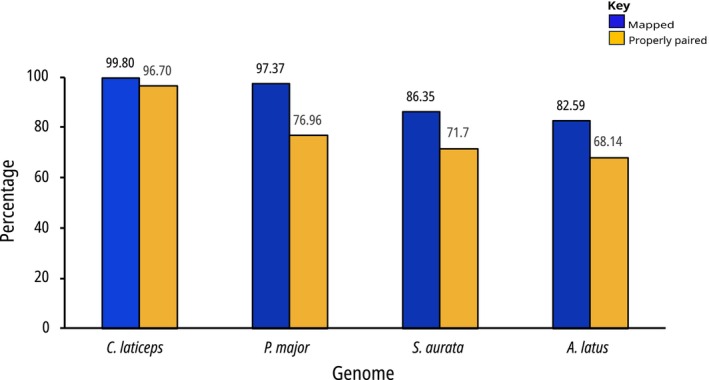
The average mapping statistics of ten samples across *C. laticeps* , *P. major* , *S. aurata* and *A. latus* genomes. Genomes are arranged on the x‐axis according to their phylogenetic distance relative to *C. laticeps* , with the most distant genome, *A. latus* , located at the end of the *x*‐axis. Blue represents the percentage of mapped reads, while orange indicates the percentage of properly paired reads, with the corresponding percentage values displayed on top of each bar.

As expected, on average, more reads were mapped and properly paired to the scaffold‐level genome of red roman (99.80% and 96.70%) than to that of the other sparids' genomes (Figure 4). Furthermore, the success of mapping correlated well with phylogenetic relatedness levels, with the percentages decreasing with an increase in evolutionary relatedness: *P. major* (97.37% and 76.96%, average mitogenome pairwise genetic distance *p* = 1.90), *S. aurata* (86.35% and 71.70%, *p* = 2.54) and *A. latus* (82.59% and 68.14%, *p* = 2.63). In this case, evolutionary relatedness proved to be a better indicator for mapping success despite the variable assembly levels of the genomes, with the *P. major* scaffold‐level genome exhibiting a larger proportion of mapped reads than the chromosome‐level genomes of *A. latus* and *S. aurata* (Figure 4). Although the number of reads mapped was very similar between red roman and *P. major* scaffold‐level genomes, a greater disparity was observed for the mapping of properly paired reads. As before, the scaffold‐level genome of red roman outperformed any of the remaining genomes. Kruskal‐Wallis test revealed significant differences in percentages for mapped reads (*p* = 2.07e^‐07^, Table S2A) and properly paired reads (*p* < 3.77e^‐07^, Table S2B) across the four genomes. For the percentage of mapped reads, Dunn's test revealed significant pairwise differences between the genomes relative to *C. laticeps* , with notable results including *C. laticeps* vs. *S. aurata* (*p* = 3.90e^‐04^) and *C. laticeps* vs. *A. latus* (*p* = 4.57e^‐07^). However, no significant difference was found for *C. laticeps* vs. *P. major* (*p* = 0.14). Additionally, the Wilcoxon test indicated that the differences between MPA and non‐MPA were not significant across all genomes (Table S2A). Considering the percentage of properly paired reads, the differences between genomes relative to *C. laticeps* were also significant across all genomes, including *P. major* , and as expected, no differences were observed between MPA and non‐MPA regions across genomes (Table S2B).

Similar to what was observed for mapping, scaffold‐level genomes outperformed chromosome‐level genomes in SNP calling, with the more distantly related *A. latus* showing the fewest total records (Table 1). However, before filtering, the *P. major* genome generated more records (SNPs and INDELS) than the *C. laticeps* genome (19,129,640 vs. 17,805,426, respectively). After filtering to retain only high‐quality SNPs, the same pattern was obtained as for the mapping results, with the scaffold‐level *C. laticeps* genome outperforming all other genomes by retaining more SNPs (Table 1). Variant filtering substantially reduced the number of SNPs across all genomes, with percentage reductions that ranged from about 92% for the *C. laticeps* , increasing with the phylogenetic distance to nearly 100% for the *S. aurata* and *A. latus* genomes (Table 2). The *S. aurata* and *A. latus* datasets experienced the highest fold reduction of more than 250 times compared to the *C. laticeps* and *P. major*, which were below 31 times (Table 2).

**TABLE 1:** Comparison of VCF files statistics in terms of number of records, number of SNPs, number of INDELs, number of multiallelic sites and number of multiallelic SNP sites before and after filtering across the scaffold‐level *C. laticeps* , *P. major* and chromosome‐level *S. aurata* and *A. latus* genomes.

Genome	VCF statistics					
	Before filtering					
	No. of samples	No. of records	No. of SNPs	No. of indels	No. of multiallelic sites	No. of multiallelic SNP sites
*C. laticeps*	10	17,805,426	15,899,724	1,905,702	458,558	165,113
*P. major*	10	19,129,640	16,682,362	2,447,278	276,520	111,770
*S. aurata*	10	8,230,567	7,117,179	1,113,388	67,699	20,179
*A. latus*	10	7,209,538	6,239,255	970,283	57,223	16,453
After filtering (DP ≥ 3, biallelic, no missing data, MAF ≥ 0.2, het ≤ 0.9, LD ≥ 0.8)						
*C. laticeps*	10	1,198,751	1,198,751	0	0	0
*P. major*	10	630,821	630,821	0	0	0
*S. aurata*	10	26,886	26,886	0	0	0
*A. latus*	10	22,371	22,371	0	0	0

Abbreviations: DP, SNP depth; HET, heterozygosity excess; INDELs, insertion and deletion mutations; LD, linkage disequilibrium *r* ^2^ value; MAF, Minor Allele Frequency.

**TABLE 2:** Comparison of VCF files statistics in terms of percentage reduction and fold reduction before and after filtering across the scaffold‐level *C. laticeps* , *P. major* and chromosome‐level *S. aurata* and *A. latus* genomes.

Genome	Statistic	Before filtering	After filtering	% Reduction	Fold reduction
*C. laticeps*	Records	17,805,426	1,198,751	93.27	14.85
	SNPs	15,899,724	1,198,751	92.46	13.26
*P. major*	Records	19,129,640	630,821	96.70	30.32
	SNPs	16,682,362	630,821	96.22	26.44
*S. aurata*	Records	8,230,567	26,886	99.67	306.2
	SNPs	7,117,179	26,886	99.62	264.8
*A. latus*	Records	7,209,538	22,371	99.69	322.3
	SNPs	6,239,255	22,371	99.64	278.8

### 3.3 Estimates of Genetic Indicator Metrics

**FIGURE 5:**
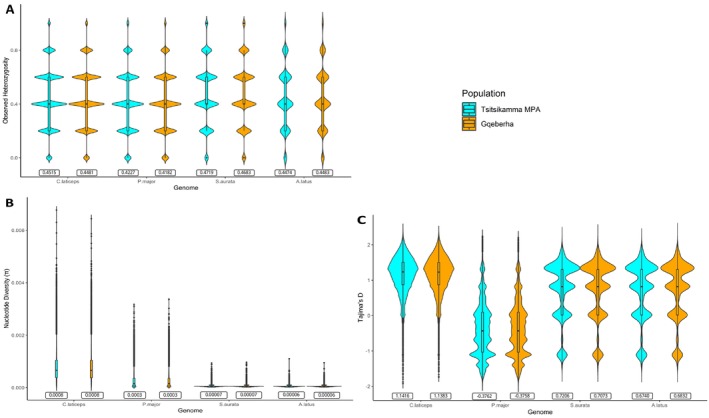
A side‐by‐side comparison of diversity metrics between Tsitsikamma MPA and Gqeberha fishing ground across the four genomes. 5A, 5B and 5C subsequently represent *H*_O_, π and Tajima's *D* with the average of each matric per population on the rectangular boxes beneath the violins.

Although the mapping and after‐filtering statistics (Figure 4; Tables 1 and 2) showed a similar trend of decline across the genomes relative to the phylogenetic distance from *C. laticeps* to *A. latus* , there was no such obvious trend with estimates of observed heterozygosity (*H*_O_) between the two sampling sites across the genomes (*H*_O_, Figure 5A). In fact, the highest average *H*_O_ was observed not for samples mapped to the *C. laticeps* genome (*H*_O_ = 0.45 for MPA and non‐MPA), but for samples mapped to *S. aurata* (*H*_O_ = 0.47 for MPA and non‐MPA). In addition, except for samples mapped to *A. latus* , estimates of *H*_O_ were slightly higher in the MPA than in the non‐MPA area (Figure 5A), but these comparisons were only statistically significant for the comparisons of samples mapped to *C. laticeps* and *P. major* . The across‐genomes (*p* < 2.20e^‐16^) and genome‐pairwise comparisons of *H*_O_ relative to *the C. laticeps* were all significant (Table S3A). When *H*_O_ was estimated across the genomes without assigning the samples to the two sampling sites, *S. aurata* again yielded the highest average *H*_O_ (0.47), followed by *C. laticeps* (0.45), *A. latus* (0.45) and *P. major* (0.42) (Table S4).

In contrast, estimated π levels were higher for samples mapped to the *C. laticeps* genome (π = 0.0008 in both Tsitsikamma MPA and Gqeberha), than for any of the other genomes (Figure 5B), and the differences were statistically significant (*p* < 2.20e^‐16^; Table S3B). The observed decrease in nucleotide diversity was proportional to an increase in phylogenetic distance, with *A. latus* showing the lowest level of π (Figure 5B). No significant differences were observed between MPA and non‐MPA samples for any of the genomes. The trend of decline of π with phylogenetic distance relative to the *C. laticeps* was also observed for the dataset that was not divided into sampling sites (Table S3).

The major difference in results comes from the estimation of Tajima's *D* (Figure 5C). Here, estimates were positive for all species, except *P. major* (*D* = −0.38 for MPA and non‐MPA), and samples mapped to *C. laticeps* had higher values (*D* = 1.14) than those for *S. aurata* (*D* = 0.72 for MPA and 0.71 for non‐MPA) or *A. latus* (*D* = 0.67 for MPA and 0.68 for non‐MPA). Although Tajima's *D* was significantly different across the four genomes (*p* < 2.20e^‐16^), genome‐pairwise and MPA vs. non‐MPA comparisons were all not significant (Table S3C). Tajima's *D* estimated from datasets not divided into the two sampling sites for the four genomes also showed a similar trend to the reported (Table S4). When these three estimates (*H*_O_, π, and Tajima's *D*) were estimated genome‐wide across the individual genomes from a dataset not filtered for LD, no major deviations were observed from the patterns here reported (Figure 5).

**FIGURE 6:**
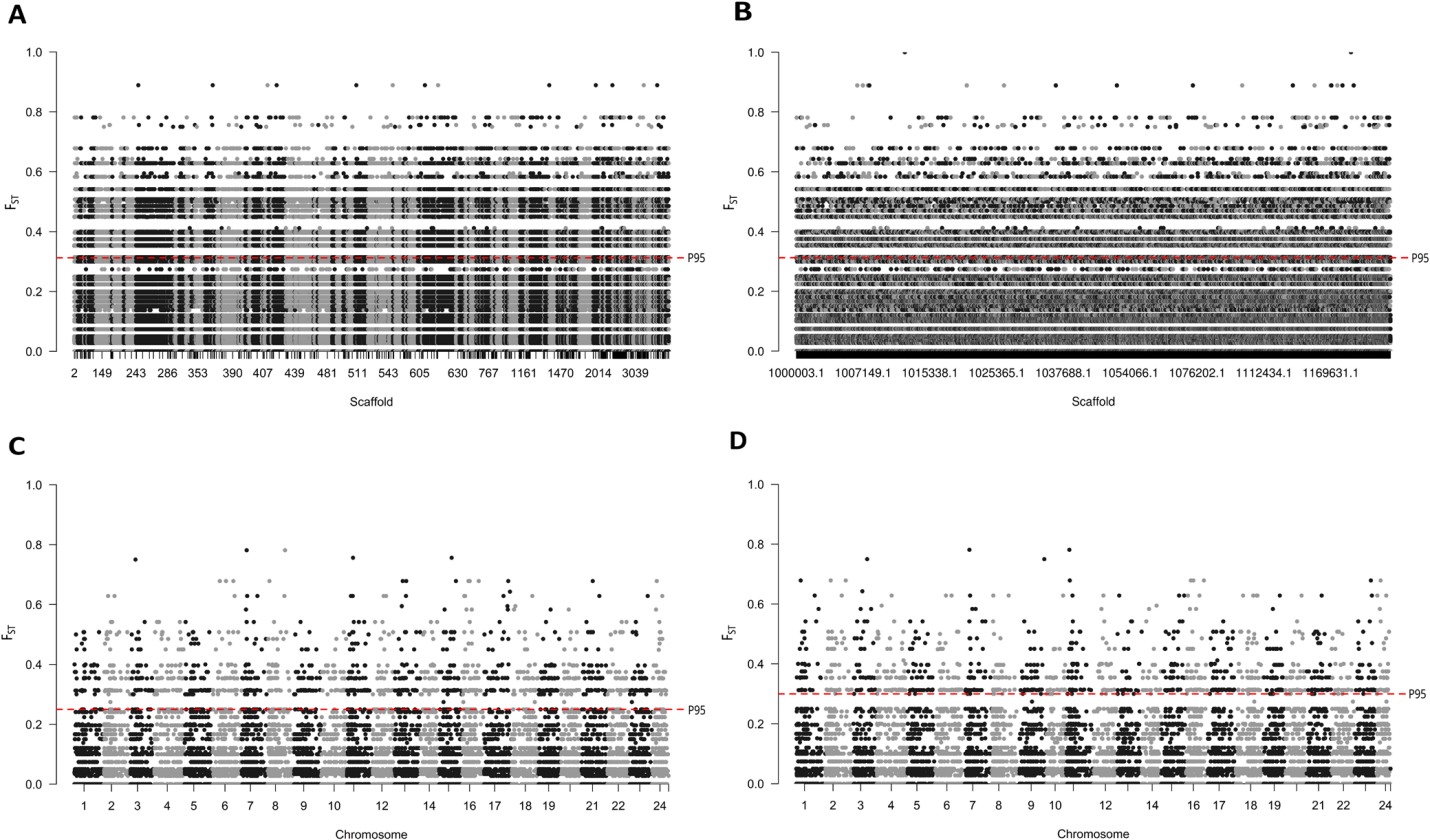
The genome‐wide *F*_ST_ distribution between Tsitsikamma MPA and Gqeberha exploited area across four genomes with a red dotted line depicting the 95^th^ percentile mark. 6A *C. laticeps* , 6B *P. major* , 6C *S. aurata* and 6D *A. latus* .

The overall (Figure 6) and average (Table 3) *F*_ST_ estimates of genetic differentiation did not show strong genome‐wide differences across the four genomes (Figure 6), but the differences were still statistically significant (*p* = 1.12e^‐11^, Table S3D). Samples mapped to *S. aurata* showed a slightly higher average *F*_ST_ between MPA and non‐MPA areas (Table 3). The *C. laticeps* dataset achieved a greater number of outliers above the 95^th^ percentile (although the average was lower) than any other genome, and this decreased (but with increasing values) with phylogeny to *A. latus* . When *F*_ST_ was assessed for the dataset not filtered for LD, more outliers above the 95th percentile (number also decreased phylogenetically from *C. laticeps* to *A. latus* ) that reached higher *F*_ST_ values were identified (Table S5; Figure S5).

**TABLE 3:** Pairwise *F*_ST_ statistics summary for the *C. laticeps* , *P. major* , *S. aurata* and *A. latus* genomes depicting the minimum, maximum, average values and the count of outliers above the 95^th^ percentile.

Genome	*F*_ST_			No. of outliers above the 95^th^ percentile	Average outliers above the 95^th^ percentile
	Min	Max	Average		
*C. laticeps*	−0.25	0.8889	−0.0086	44,373	0.0370
*P. major*	−0.25	1	−0.0087	23,332	0.0370
*S. aurata*	−0.25	0.7813	−0.0078	1271	0.0473
*A. latus*	−0.25	0.7813	−0.0087	1090	0.0487

Of the methods tested to estimate *N*_e_ when the individuals from MPA and non‐MPA were combined into one population, only the Heterozygote Excess method did not converge, except for mapping to *S. aurata* (Table 4). Overall, the estimates of the LD method were higher for *P. major* compared to *C. laticeps* , followed by *S. aurata* and *A. latus* (Table 4). The Molecular Coancestry method showed a different trend to that of the LD method, with *A. latus* producing higher *N*_e_ estimations (38.20), followed by *C. laticeps* (18.80), *S. aurata* (18.70) and *P. major* (14.00). When *N*_e_ was estimated on the separate populations for MPA and non‐MPA, only the Molecular Coancestry method converged across the two populations across all genomes, whereas the other methods did not converge except for the Heterozygote Excess method for the *S. aurata* genome (Table S6). These results demonstrate how small sample sizes can bias the estimation of *N*_e_, even when there are thousands of loci.

**TABLE 4:** The comparison of *N*_e_ estimates and the confidence interval (CI) from the Linkage disequilibrium, Heterozygote excess and Molecular coancestry methods across the *C. laticeps* , *P. major* , *S. aurata* and *A. latus* genomes.

Genome	Method	Sample size	Estimate of *N*_*e*_	95% CI
*C. laticeps*	Linkage disequilibrium	10	1371.5	1362.7–1379.7
	Heterozygote excess	10	Infinite	Infinite
	Molecular Coancestry	10	18.8	18.4–19.2
*P. major*	Linkage disequilibrium	10	1672.6	1655.1–1689.8
	Heterozygote excess	10	Infinite	Infinite
	Molecular Coancestry	10	14	13.7–14.3
*S. aurata*	Linkage disequilibrium	10	1647.7	1488.3–1845.6
	Heterozygote excess	10	20.1	17.5–23.6
	Molecular Coancestry	10	18.7	17.3–20.0
*A. latus*	Linkage disequilibrium	10	1640.8	1454.3–1882.2
	Heterozygote excess	10	Infinite	Infinite
	Molecular Coancestry	10	38.2	31.8–45.2

## 4 Discussion

The extent of biodiversity that has already been lost as a result of human activities, environmental and climate changes is unquantified, and yet there is a continuation of loss within and among species on a global scale at a rate faster than we can document (Hoban et al. 2022, 2013; Pinto et al. 2024; Shaw et al. 2025). Genetic diversity is a major component of biodiversity, and there is an urgent need to assess genetic indicators, i.e., metrics that quantify three major components of the evolutionary potential of species: genetic diversity, genetic differentiation, and effective population size (Hoban et al. 2022, 2021). Recent work, including Akopyan et al. (2024), Rhie et al. (2021) and Thorburn et al. (2023), suggests that the choice of reference genome (in terms of quality, assembly level and the phylogenetic relationship of the assembly to the samples) can strongly influence the estimates of various genetic indicators. Our results build on this previous work, demonstrating that this phenomenon applies to endangered marine fishes as well.

### 4.1 A Reference Genome for the Red Roman

The quality of available genomes is critically important in conservation biology and must be addressed. In light of the ongoing sixth mass extinction, high‐quality genomes are crucial for successful conservation efforts as they provide a more comprehensive characterisation of genomic information compared to fragmented genomes (Formenti et al. 2022). Published genomes for most non‐model species are of concern due to the limitations of early sequencing technologies, such as first‐generation short‐read sequencing, and the lack of sufficient genetic resources (Rice and Green 2019; Song and Mitchell‐Olds 2011; Wattad et al. 2024). In this paper, we highlight the importance of selecting the right genomic resources for accurate genetic analyses and present a high‐quality, annotated *C. laticeps* genome obtained using long‐read Oxford Nanopore sequencing technology, which has a higher long‐read sequencing accuracy than other technologies (Guiglielmoni et al. 2022; Sereika et al. 2022).

The N50 scaffold size of *C. laticeps* (5.89 Mb) demonstrated a significantly improved contiguous assembly compared to the relatively fragmented assembly of *P. major* (4.6 kb). However, it remains inferior to the more advanced chromosomal assemblies of *S. aurata* and *A. latus* , as anticipated, except for the L50 metric (Table S7). Furthermore, the coverage depth of the *C. laticeps* genome was over three times greater than that of other available genomes for sparids, including *P. major* , *S. aurata* , and two times greater than that of *A. latus* , used here for comparison. This enhanced genome‐wide coverage depth provided a robust foundation for precise genotyping across the entire genome (Lloret‐Villas et al. 2021). In addition, the high BUSCO completeness, with low percentages of duplication, fragmentation and missingness, suggests a relatively complete genome, albeit not yet assembled at the chromosome level. As expected, a better and species‐specific genome resulted in higher mapping quality, a larger number of mapped reads, and, most importantly, a larger number of properly paired mapped reads, even when compared to *S. aurata* and *A. latus* genomes that are assembled to a chromosome level. Although the percentage of the mapped reads was not significantly different between *C. laticeps* and *P. major* , the difference in the percentages of properly paired reads was very large and statistically significant. These results support the hypothesis that it is better to have a good‐quality scaffolded reference assembly of the species of interest than a chromosome‐level genome of a related species.

A high‐quality reference genome coupled with proper annotations is essential for biodiversity conservation (Formenti et al. 2022). Understanding the gene functions relative to the environmental conditions of organisms is an essential step, and as a start, the *C. laticeps* genome underwent two rigorous rounds of annotation to optimise its configuration and enhance the discovery of critical genome‐wide features, including genes, CDS and mRNAs. During the analytical comparison of this genome with the three sparid species, only the *A. latus* (NCBI Accession GCA_904848185.1) and *S. aurata* (NCBI Accession GCA_900880675.1) genomes had been annotated. However, the annotation of the *P. major* (NCBI Accession GCA_040436345.1) genome is now available. Despite the scaffold assembly level of the *C. laticeps* , its annotations identified more genes and CDS relative to the other three sparid genomes available at the time (NCBI accession numbers GCF_040436345.1‐RS_2025_04, GCF_900880675.1‐RS_2025_07 and *A. latus* Annotation Release 100). Although this does not necessarily imply superiority of the *C. laticeps* genome to the three genomes because of different species being compared, it affirms the genome's ability to investigate the interactions between genomic features and environmental conditions more comprehensively in this species, an invaluable tool in the face of climate change, as the species is showing signs of different physiological responses to different exploitation levels (Duncan et al. 2019).

Unlike microsatellites that are usually multiallelic, SNPs are mostly biallelic, thus a substantial number of SNPs is required to accurately estimate genetic diversity within and across populations (Morin et al. 2004). Attaining a higher number of SNPs from genomic data is required for population analyses, as it enhances the statistical power of inference and has the potential to discover outliers that are subjected to selection (Allendorf and Seeb 2000; Xia et al. 2019). Although the VCF file generated from samples mapped to the *P. major* genome demonstrated a higher number of recovered records (including SNPs, INDELS, multiallelic sites and multiallelic SNP sites) through SNP calling, indicating a superior SNP recovery rate compared to the conspecific *C. laticeps* and the other two genomes, the remaining number of SNP records for *C. laticeps* after applying filters exceeded that of the *P. major* , *S. aurata* and *A. latus* genomes by more than 89%. This again underscores the advantage of a high‐quality conspecific genome, irrespective of its assembly level. This is evidence that a genome's quality and superiority should not be based solely on general SNP calling, as it may encompass a range of low‐quality and cross‐species SNPs, but rather on a carefully filtered dataset.

### 4.2 Influence of the Choice of Genome on the Estimates of Genetic Indicators for Conservation Actions in Red Roman

Based on the findings presented here, it is evident that the phylogenetic distance between the reference genome and the study species has a significant impact across the majority of metrics used as genetic indicators for conservation actions, even in highly genetically diverse marine fishes. However, this impact varied with the metrics used, being subtle for *H*_O_ and more pronounced for π and Tajima's *D*. While it has been shown that the measures of genetic diversity and variability deteriorate with increased phylogenetic distance (Prasad et al. 2022; Valiente‐Mullor et al. 2021), this is not universally the case. For example, in this study, *A. latus* exhibited findings comparable to those for *S. aurata* , and demonstrated higher diversity than *P. major* . While the differences in *H*_O_ between the *C. laticeps* genome and the other genomes (except that of *A. latus* ) were significant, their estimations were comparable in range. Despite this, samples mapped to the *C. laticeps* genome showed a wider genome‐wide distribution of heterozygosity estimates compared to the three genomes, and this is likely due to high SNP density influencing a wider scope of inference from the conspecific genome. Nucleotide diversity, in contrast, was severely underestimated by the three genomes compared to *C. laticeps* , with the more distantly related genomes showing a 10‐fold decrease in estimates. Therefore, our results suggest that the use of heterospecific genomes has the potential for a strongly biased interpretation of genetic diversity in the target species and, consequently, on the evolutionary processes that shaped extant patterns of diversity. Both heterozygosity and π quantify genetic variation and have a fundamental role in the survival, adaptability and evolution of species, and they represent varying underlying forces shaping the diversity of populations. For example, population bottlenecks that occur over a short time are unlikely to result in reduced heterozygosity; rather, they will decrease the number of alleles (and indirectly nucleotide diversity) in the population (Allendorf 1986), thus leaving an imprint in metrics such as nucleotide diversity, but not heterozygosity. In our case, if the genomes of *S. aurata* or *A. latus* had been solely used, the high *H*_O_ and low π could suggest recent changes in population sizes that were not observed when comparing metrics obtained with the *C. laticeps* genome assembly.

Similarly, results for Tajima's *D* suggest different evolutionary histories depending on which reference genome is used. While results obtained for the *C. laticeps* , *S. aurata* , and *A. latus* were all positive (although with different average values), values obtained for *P. major* were on average lower and negative, implying quite disparate evolutionary trajectories. Tajima's *D* has the potential to assess forces behind changes in the demography of populations (Tajima 1989a). If we used the −2 and 2 Tajima's *D* scale to propose the deviation of populations from neutral evolution (Eckshtain‐Levi et al. 2018; Tajima 1989a), then all genomes support neutral evolution, but different demographic events. Interestingly, these patterns disappeared when the dataset that was not filtered for LD was used, suggesting that the distribution of markers across the genome is not uniform across the four genomes. However, small sample sizes have been shown to bias the estimates of Tajima's *D* in different studies (e.g., Larsson et al. 2013; Subramanian 2016), and a larger sample size would be essential to make confident conclusions.

Another important metric assessed in this study was *F*_ST_, for which the average estimates were similar across all the genomes in both LD‐filtered and non‐filtered datasets. Despite the comparable average estimations across the genomes, the conspecific *C. laticeps* genome identified 47% more outlier loci than the more phylogenetically related *P. major* and more than 97% than for both *S. aurata* and *A. latus* . This suggests that the conspecific genome has greater potential for the detection of more SNPs that could be associated with evolutionary forces that drive divergence between populations. Similar findings were observed by Thorburn et al. (2023) for stickleback, where the use of conspecific genomes from the same region as the study samples increased the ability to detect outlier loci linked with local adaptation patterns. For marine species, where local adaptation can persist in the presence of gene flow, our results show that conspecific genomes are fundamental in detecting subtle levels of differentiation and the action of natural selection.

While it is obvious that genetic diversity and differentiation play crucial roles in conservation, the importance of *N*_e_ must not be overlooked. This genetic metric evaluates the extinction risk of populations and greatly influences their resilience in the face of environmental changes (Hoban et al. 2021). It influences the level of genetic diversity lost due to genetic drift and evaluates the degree of inbreeding depression, which can impact the fitness and adaptation of individuals (Crow 2017). Franklin (1980) proposed a 50/500 rule, suggesting a minimum *N*_e_ of 50 to reduce short‐term inbreeding and 500 to maintain long‐term genetic diversity needed for adaptation. Both the finite and infinite estimations of *N*_*e*_ using the LD and Heterozygosity Excess methods across the *C. laticeps* , *P. major* and *A. latus* genomes should be sufficient for the persistence of the red roman populations, but these methods are known to be unreliable for small sample sizes such as this (England et al. 2006; Luikart and Cornuet 1999; Pudovkin et al. 1996; Saura et al. 2015; Waples 2006), and thus a larger sample size would significantly strengthen the significance of results observed in this study using this method. The Molecular Coancestry method is less reliant on sample size than the other two methods (Nomura 2008), therefore, it reliably addressed the inferred small *N*_e_ (below 40 for all genomes) in the small sample size of this study. Although the final numbers should not be taken at face value due to the possibility of overlapping cohorts as well as small sample sizes, our results suggest that using heterospecific genomes upward biases the estimates of *N*_e_, regardless of phylogenetic distance. In all cases, estimates using the *C. laticeps* genome assembly were generally smaller than those obtained with other genomes. Therefore, our findings suggest that caution should be exercised when calculating *N*_e_ based on heterospecific genomes, not because it might downward bias the estimates, but because it might overestimate them.

This study identifies differences in the estimations of genetic metrics used to inform conservation actions for natural populations and suggests that the phylogenetic relationship between the study species and the reference genome is critical. For example, if we had only mapped the sample reads to *P. major* , which would have been the case in the absence of a conspecific genome as this species is most closely related to *C. laticeps* that is published, we would have assumed that *C. laticeps* had lower *H*_O_ and Tajima's *D*, higher π and higher *N*_e_. Vice versa, if the sample reads were mapped to *S. aurata* , which during that time was the closest published relative to *C. laticeps* available at the chromosome‐level, we would have thought that *C. laticeps* had higher *H*_O_ and Tajima's *D*, and lower π than inferred in this study. This interpretation was different across all four genomes and the various metrics assessed. Therefore, it is recommended that a conspecific reference genome (even at the scaffold level) be used in population genetics analyses to eliminate bias in the measures of genetic metrics that can be heterospecific reference genome based.

Regardless of the assembly level of a genome, be it at the scaffold or the chromosome level, the sequencing quality and phylogenetic distance of the reference genome to the study species are important considerations in shaping the downstream population genomics analyses, even for marine fishes. This paper highlights the importance of conspecific reference genome in conservation, and that it is not a concept that can merely be concluded based on an individual genetic metric, but rather the joint analyses of various metrics that provide information on the diversity and variability of populations. More important questions that are species‐specific in the scope of biodiversity may arise for effective conservation management of our threatened species in the near future, and annotated conspecific reference genomes stand a better chance of addressing them and minimising the conservation genomics gap. Although this paper could not confidently resolve the differences between Tsitsikamma MPA and Gqeberha, mostly due to the small sample size and/or possibly the lack of differentiation between these areas, it consistently demonstrated that the phylogenetic relationship of the genome to the study samples strongly influences the estimations of genetic metrics, highlighting the importance of generating such a genome for threatened and exploited marine fish.

## Funding

This work was supported by the National Research Foundation, ACEP200414513037, MND210527604276, PMDS240527221246 and Department of Science and Innovation, South Africa.

## Conflicts of Interest

The authors declare no conflicts of interest.

## Supporting information


**Figure S1.** Genome coverage depth and GC content across *C. laticeps* , *P. major* , *S. aurata* and *A. latus* genomes.
**Figure S2.** The Blobtools Mollusc hit Blast results from NCBI.
**Figure S3.** The first multi‐locus phylogenetic tree of seabreams (Santini et al. 2014). The three NCBI genomes and the Red Roman are boxed in red rectangles.
**Figure S4.** A side‐by‐side comparison of diversity metrics (from a VCF file not filtered for LD) between Tsitsikamma MPA and Gqeberha fishing ground across the four genomes. S4A, S4B and S4C subsequently represent *H*_O_, π and Tajima's *D* with the average of each matric per population on the rectangular boxes beneath the violins.
**Figure S5.** The comparisons of π between Tsitsikamma MPA and Gqeberha across the genome, using a VCF file that is not filtered for LD.
**Figure S6.** The comparisons of Tajima's *D* between Tsitsikamma MPA and Gqeberha across the genome, using a VCF file that is not filtered for LD.
**Figure S7.** The genome‐wide *F*_ST_ distribution from a non‐LD filtered dataset between Tsitsikamma MPA and Gqeberha exploited area across four genomes, with a red dotted line depicting the 95‐percentile mark. S7A *C. laticeps* , S7B *P. major* , S7C *S. aurata* and S7D *A. latus* .
**Table S1.** The red roman Blastn results against the mitogenome dataset of *P. major* .
**Table S2.** Showing Wilcoxon, Kruskal–Wallis and Dunn's statistical tests between mapped and properly paired reads across the *C. laticeps* , *P. major* , *S. aurata* and *A. latus* genomes.
**Table S3.** Showing Wilcoxon, Kruskal‐Wallis and Dunn's statistical tests of *H*_O_, π, Tajima's *D* and *F*_ST_ across the *C. laticeps* , *P. major* , *S. aurata* and *A. latus* genomes.
**Table S4.** The comparison of the average estimates of *H*_O_, π and Tajima's *D* between the dataset filtered and not filtered for LD across *C. laticeps* , *P. major* , *S. aurata* and *A. latus* .
**Table S5.** The *F*_ST_ statistics summary for the non‐LD filtered dataset across the *C. laticeps* , *P. major* , *S. aurata* and *A. latus* genomes depicting the minimum, maximum, average, number and average of outliers above the 95th percentile.
**Table S6.** The comparison of *N*_e_ estimates and the confidence interval (CI) from the Linkage disequilibrium, Heterozygote excess and Molecular coancestry methods between Tsitsikamma MPA and Gqeberha across the *C. laticeps* , *P. major* , *S. aurata* and *A. latus* genomes.
**Table S7.** Genome assembly information for *C. laticeps* , *P. major* , *S. aurata* and *A. latus* .

## Data Availability

The assembly sequencing report, annotations, raw fastq files and scripts are uploaded to the Marine Genomics LineFish_Evolution repository in Github (https://github.com/marine‐genomics/LineFish_Evolution), and the genome assembly + annotation and raw reads to the European Nucleotide Archive (Project PRJEB111808, and PRJEB112359, respectively).
