# Supplementary material for: Conspecific Scaffold‐Level Genome Assembly Outperforms Heterospecific Chromosome‐Level Assemblies for Assessing Genetic Indicators in a Threatened Marine Fish
Source: Evol Appl. 2026 May 5;19(5):e70247. doi: 10.1111/eva.70247 (PMC13144753; doi:10.1111/eva.70247)
Supplement: Supplementary file 1 — Figure S1. Genome coverage depth and GC content across C. laticeps , P. major , S. aurata and A. latus genomes. Figure S2. The Blobtools Mollusc hit Blast results from NCBI. Figure S3. The first multi‐locus phylogenetic tree of seabreams (Santini et al. 2014). The three NCBI genomes and the Red Roman are boxed in red rectangles. Figure S4. A side‐by‐side comparison of diversity metrics (from a VCF file not filtered for LD) between Tsitsikamma MPA and Gqeberha fishing ground across the four genomes. S4A, S4B and S4C subsequently represent H O, π and Tajima's D with the average of each matric per population on the rectangular boxes beneath the violins. Figure S5. The comparisons of π between Tsitsikamma MPA and Gqeberha across the genome, using a VCF file that is not filtered for LD. Figure S6. The comparisons of Tajima's D between Tsitsikamma MPA and Gqeberha across the genome, using a VCF file that is not filtered for LD. Figure S7. The genome‐wide F ST distribution from a non‐LD filtered dataset between Tsitsikamma MPA and Gqeberha exploited area across four genomes, with a red dotted line depicting the 95‐percentile mark. S7A C. laticeps , S7B P. major , S7C S. aurata and S7D A. latus . Table S1. The red roman Blastn results against the mitogenome dataset of P. major . Table S2. Showing Wilcoxon, Kruskal–Wallis and Dunn's statistical tests between mapped and properly paired reads across the C. laticeps , P. major , S. aurata and A. latus genomes. Table S3. Showing Wilcoxon, Kruskal‐Wallis and Dunn's statistical tests of H O, π, Tajima's D and F ST across the C. laticeps , P. major , S. aurata and A. latus genomes. Table S4. The comparison of the average estimates of H O, π and Tajima's D between the dataset filtered and not filtered for LD across C. laticeps , P. major , S. aurata and A. latus . Table S5. The F ST statistics summary for the non‐LD filtered dataset across the C. laticeps , P. major , S. aurata and A. latus genomes depicting the minimum, maximum, av [file EVA-19-e70247-s001.docx]

Count

| Genome | Coverage depth | GC percentage |
| --- | --- | --- |
| C. laticeps | 161 | 41.06 |
| P. major | 16 | 40.5 |
| S. aurata | 56 | 42 |
| A. latus | 60 | 42 |

**Suppl. Fig. 1**. Genome coverage depth and GC content across *C. laticeps*, *P. major*, *S. aurata*, and *A. latus* genomes.

**Suppl. Fig. 2.** The Blobtools Mollusc hit Blast results from NCBI

**Suppl. Fig. 3**. The first multi-locus phylogenetic tree of seabreams (Santini et al., 2014). The three NCBI genomes and the Red Roman are boxed in red rectangles.

**Suppl. Fig. 4**. A side-by-side comparison of diversity metrics (from a VCF file not filtered for LD) between Tsitsikamma MPA and Gqeberha fishing ground across the four genomes. S4A, S4B, and S4C subsequently represent *H*_O_, π, and Tajima’s *D* with the average of each matric per population on the rectangular boxes beneath the violins.

**Suppl. Fig. 5**. The comparisons of π between Tsitsikamma MPA and Gqeberha across the genome, using a VCF file that is not filtered for LD.

**Suppl. Fig. 6.** The comparisons of Tajima’s *D* between Tsitsikamma MPA and Gqeberha across the genome, using a VCF file that is not filtered for LD.

**Suppl. Fig. 7**. The genome-wide *F*_ST_ distribution from a non-LD filtered dataset between Tsitsikamma MPA and Gqeberha exploited area across four genomes, with a red dotted line depicting the 95-percentile mark. S7A *C. laticeps*, S7B *P. major*, S7C *S. aurata*, and S7D *A. latus.*

**Suppl. Table 1.** The red roman Blastn results against the mitogenome dataset of *P. major*

| **qseqid** | **sseqid** | **pident** | **length** | **mismatch** | **gapopen** | **qstart** | **qend** | **sstart** | **send** | **evalue** | **bitscore** |
| --- | --- | --- | --- | --- | --- | --- | --- | --- | --- | --- | --- |
| **contig_2019** | **NC_003196.1** | **92.764** | **4505** | **319** | **7** | **1** | **4503** | **157** | **4656** | **0** | **6630** |
| **contig_2019** | **NC_003196.1** | **95.946** | **148** | **5** | **1** | **1** | **148** | **16885** | **17031** | **2.40E-64** | **237** |
| **contig_2018** | **NC_003196.1** | **87.213** | **11011** | **1397** | **8** | **6** | **11009** | **4673** | **15679** | **0** | **13484** |
| **contig_2018** | **NC_003196.1** | **84.166** | **941** | **128** | **16** | **11329** | **12257** | **16100** | **17031** | **0** | **973** |
| **contig_2018** | **NC_003196.1** | **96.222** | **450** | **16** | **1** | **11954** | **12403** | **1** | **449** | **0** | **732** |

**Suppl. Table 2**. Showing Wilcoxon, Kruskal-Wallis, and Dunn’s statistical tests between mapped and properly paired reads across the *C. laticeps*, *P. major*, *S. aurata*, and *A. latus* genomes.

| **Analyses** | **Comparison Type** | **Groups compared** | **Test Type** | **P-value** (BH) |
| --- | --- | --- | --- | --- |
| A. Mapped reads | Between the Tsitsikamma MPA and Gqeberha | *C. laticeps* | Wilcoxon | 1 |
|  |  | *P. major* | Wilcoxon | 0.6905 |
|  |  | *S. aurata* | Wilcoxon | 0.8413 |
|  |  | *A. latus* | Wilcoxon | 1 |
|  | Across genomes | Four genomes | Kruskal-Wallis | 2.07e-07 |
|  | Genome pairwise | *C. laticeps* vs *P. major* | Dunn | 1.347805e-01 |
|  |  | *C. laticeps* vs *S. aurata* | Dunn | 3.904443e-04 |
|  |  | *C. laticeps* vs *A. latus* | Dunn | 4.575102e-07 |
|  |  | *P. major* vs *S. aurata* | Dunn | 3.780085e-02 |
|  |  | *P. major* vs *A. latus* | Dunn | 3.038319e-04 |
|  |  | *S. aurata* vs *A. latus* | Dunn | 1.212370e-01 |
| B. Properly paired reads | Between the Tsitsikamma MPA and Gqeberha | *C. laticeps* | Wilcoxon | 0.4206 |
|  |  | *P. major* | Wilcoxon | 0.6905 |
|  |  | *S. aurata* | Wilcoxon | 0.8413 |
|  |  | *A. latus* | Wilcoxon | 0.8413 |
|  | Across genomes | Four genomes | Kruskal-Wallis | 3.768e-07 |
|  | Genome pairwise | *C. laticeps* vs *P. major* | Dunn | 4.825912e-02 |
|  |  | *C. laticeps* vs *S. aurata* | Dunn | 2.644405e-04 |
|  |  | *C. laticeps* vs *A. latus* | Dunn | 3.718103e-07 |
|  |  | *P. major* vs *S. aurata* | Dunn | 9.031939e-02 |
|  |  | *P. major* vs *A. latus* | Dunn | 2.145113e-03 |
|  |  | *S. aurata* vs *A. latus* | Dunn | 1.357176e-01 |

**Suppl. Table 3**. Showing Wilcoxon, Kruskal-Wallis, and Dunn’s statistical tests of *H*_O_, π, Tajima’s *D*, and *F*_ST_ across the *C. laticeps*, *P. major*, *S. aurata*, and *A. latus* genomes.

| **Analyses** | **Comparison Type** | **Groups compared** | **Test Type** | **P-value** (BH) |
| --- | --- | --- | --- | --- |
| A. *H*_O_ | Between the Tsitsikamma MPA and Gqeberha | *C. laticeps* | Wilcoxon | < 2e^-16^ |
|  |  | *P. major* | Wilcoxon | < 2e^-16^ |
|  |  | *S. aurata* | Wilcoxon | 0.06192 |
|  |  | *A. latus* | Wilcoxon | 0.7337 |
|  | Across genomes | Four genomes | Kruskal-Wallis | < 2.2e^-16^ |
|  | Genome pairwise | *C. laticeps* vs *P. major* | Dunn | 0 |
|  |  | *C. laticeps* vs *S. aurata* | Dunn | 6.503580e^-82^ |
|  |  | *C. laticeps* vs *A. latus* | Dunn | 5.451203e^-03^ |
|  |  | *P. major* vs *S. aurata* | Dunn | 0 |
|  |  | *P. major* vs *A. latus* | Dunn | 1.343923e^-135^ |
|  |  | *S. aurata* vs *A. latus* | Dunn | 9.059053e^-52^ |
| B. π | Between the Tsitsikamma MPA and Gqeberha | *C. laticeps* | Wilcoxon | 0.7108 |
|  |  | *P. major* | Wilcoxon | 0.3589 |
|  |  | *S. aurata* | Wilcoxon | 0.4881 |
|  |  | *A. latus* | Wilcoxon | 0.4348 |
|  | Across genomes | Four genomes | Kruskal-Wallis | < 2.2e^-16^ |
|  | Genome pairwise | *C. laticeps* vs *P. major* | Dunn | 0 |
|  |  | *C. laticeps* vs *S. aurata* | Dunn | 0 |
|  |  | *C. laticeps* vs *A. latus* | Dunn | 0 |
|  |  | *P. major* vs *S. aurata* | Dunn | 0 |
|  |  | *P. major* vs *A. latus* | Dunn | 0 |
|  |  | *S. aurata* vs *A. latus* | Dunn | 0.01419977 |
| C. Tajima’s *D* | Between the Tsitsikamma MPA and Gqeberha | *C. laticeps* | Wilcoxon | 0.1125 |
|  |  | *P. major* | Wilcoxon | 0.6471 |
|  |  | *S. aurata* | Wilcoxon | 0.2643 |
|  |  | *A. latus* | Wilcoxon | 0.4015 |
|  | Across genomes | Four genomes | Kruskal-Wallis | < 2.2e^-16^ |
|  | Genome pairwise | *C. laticeps* vs *P. major* | Dunn | 0 |
|  |  | *C. laticeps* vs *S. aurata* | Dunn | 0 |
|  |  | *C. laticeps* vs *A. latus* | Dunn | 0 |
|  |  | *P. major* vs *S. aurata* | Dunn | 0 |
|  |  | *P. major* vs *A. latus* | Dunn | 0 |
|  |  | *S. aurata* vs *A. latus* | Dunn | 3.129752e^-06^ |
| D. *F*_ST_ | Across genomes | Four genomes | Kruskal-Wallis | 1.118e-11 |
|  | Genome pairwise | *C. laticeps* vs *P. major* | Dunn | 2.137049e^-06^ |
|  |  | *C. laticeps* vs *S. aurata* | Dunn | 1.803197e^-06^ |
|  |  | *C. laticeps* vs *A. latus* | Dunn | 6.270082e^-01^ |
|  |  | *P. major* vs *S. aurata* | Dunn | 6.261998e^-09^ |
|  |  | *P. major* vs *A. latus* | Dunn | 1.274374e^-01^ |
|  |  | *S. aurata* vs *A. latus* | Dunn | 4.255728e^-03^ |

**Suppl. Table 4.** The comparison of the average estimates of *H*_O_, π, and Tajima’s *D* between the dataset filtered and not filtered for LD across *C. laticeps*, *P. major*, *S. aurata*, and *A. latus*.

| **Genome** | **LD or non-LD filtered dataset** | **Average estimate across genomes** | | |
| --- | --- | --- | --- | --- |
|  |  | ***H*_O_** | **π** | **Tajima’s *D*** |
| *C. laticeps* | LD | 0.449755 | 0.000761819 | 1.886288 |
|  | Non-LD | 0.424619 | 0.00378276 | -1.110518 |
| *P. major* | LD | 0.420401 | 0.000247923 | -0.4754859 |
|  | Non-LD | 0.418075 | 0.000351632 | -0.8544739 |
| *S. aurata* | LD | 0.470133 | 6.48123e-05 | 1.064162 |
|  | Non-LD | 0.45908 | 0.000115794 | -0.905708 |
| *A. latus* | LD | 0.447848 | 6.26522e-05 | 1.032685 |
|  | Non-LD | 0.434541 | 0.000102259 | -0.9881444 |

**Suppl. Table 5.** The *F*_ST_ statistics summary for the non-LD filtered dataset across the *C. laticeps*, *P. major*, *S. aurata*, and *A. latus* genomes depicting the minimum, maximum, average, number and average of outliers above the 95^th^ percentile.

| **Genome** | ***F*_ST_ statistics** | | | | | |
| --- | --- | --- | --- | --- | --- | --- |
|  | **Min** | **Max** | **Average** | **No. of outliers above the 95^th^ percentile** | **Average of outliers above the 95^th^ percentile** |  |
| *C. laticeps* | -0.25 | 1 | -0.0084 | 162401 | 0.0495 |  |
| *P. major* | -0.25 | 1 | -0.0087 | 26442 | 0.0374 |  |
| *S. aurata* | -0.25 | 0.8889 | -0.0071 | 3103 | 0.0488 |  |
| *A. latus* | -0.25 | 0.7813 | -0.0076 | 2227 | 0.0496 |  |

**Suppl. Table 6.** The comparison of *N*_e_ estimates and the confidence interval (CI) from the Linkage disequilibrium, Heterozygote excess, and Molecular coancestry methods between Tsitsikamma MPA and Gqeberha across the *C. laticeps*, *P. major*, *S. aurata*, and *A. latus* genomes.

| **Genome** | **Method** | **Location** | **Sample size (Harmonic Mean)** | **Estimate of N_e_** | **95% Confidence Interval** |
| --- | --- | --- | --- | --- | --- |
| *C. laticeps* | Linkage Disequilibrium | Tsitsikamma MPA | 5 | Infinite | Infinite |
|  |  | Gqeberha | 5 | Infinite | Infinite |
|  | Heterozygote Excess | Tsitsikamma MPA | 5 | Infinite | Infinite |
|  |  | Gqeberha | 5 | Infinite | Infinite |
|  | Molecular Coancestry | Tsitsikamma MPA | 5 | 17.2 | 16.6 |
|  |  | Gqeberha | 5 | 12.5 | 12 |
| *P. major* | Linkage Disequilibrium | Tsitsikamma MPA | 5 | Infinite | Infinite |
|  |  | Gqeberha | 5 | Infinite | Infinite |
|  | Heterozygote Excess | Tsitsikamma MPA | 5 | Infinite | Infinite |
|  |  | Gqeberha | 5 | Infinite | Infinite |
|  | Molecular Coancestry | Tsitsikamma MPA | 5 | 10.4 | 10.1 |
|  |  | Gqeberha | 5 | 10.1 | 9.8 |
| *S. aurata* | Linkage Disequilibrium | Tsitsikamma MPA | 5 | Infinite | Infinite |
|  |  | Gqeberha | 5 | Infinite | Infinite |
|  | Heterozygote Excess | Tsitsikamma MPA | 5 | 19.3 | 16.4 |
|  |  | Gqeberha | 5 | 23.3 | 19.2 |
|  | Molecular Coancestry | Tsitsikamma MPA | 5 | 20.5 | 18.9 |
|  |  | Gqeberha | 5 | 21.6 | 19.9 |
| *A. latus* | Linkage Disequilibrium | Tsitsikamma MPA | 5 | Infinite | Infinite |
|  |  | Gqeberha | 5 | Infinite | Infinite |
|  | Heterozygote Excess | Tsitsikamma MPA | 5 | Infinite | Infinite |
|  |  | Gqeberha | 5 | Infinite | Infinite |
|  | Molecular Coancestry | Tsitsikamma MPA | 5 | 14 | 13.1 |
|  |  | Gqeberha | 5 | 11.8 | 11 |

**Suppl. Table 7**. Genome assembly information for *C. laticeps*, *P. major*, *S. aurata*, and *A. latus*.

| **Statistic** | ***C. laticeps*** | ***P. major*** | ***S. aurata*** | ***A. latus*** |
| --- | --- | --- | --- | --- |
| Genome size (Mb) | 758.3 | 875.5 | 833.6 | 685.1 |
| Scaffold N50 | 5.89 Mb | 4.6 kb | 35.8 Mb | 30.7 Mb |
| Scaffold L50 | 36 | 35 607 | 11 | 11 |
| Contig N50 | 5.89 Mb | 2.8 kb | 2.9 Mb | 14.9 Mb |
| Contig L50 | 40 | 60 234 | 75 | 18 |
| Publication date | 2025 | 2017 | 2021 | 2020 |
